# Development and Psychometric Properties of the DASS-Youth (DASS-Y): An Extension of the Depression Anxiety Stress Scales (DASS) to Adolescents and Children

**DOI:** 10.3389/fpsyg.2022.766890

**Published:** 2022-04-14

**Authors:** Marianna Szabo, Peter F. Lovibond

**Affiliations:** ^1^School of Psychology, The University of Sydney, Darlington, NSW, Australia; ^2^School of Psychology, University of New South Wales, Kensington, NSW, Australia

**Keywords:** depression, anxiety, stress, child, adolescent, DASS, DASS – 21, DASS-Y

## Abstract

The Depression Anxiety Stress Scales (DASS; Lovibond and Lovibond, 1995b) is a set of psychometrically sound scales that is widely used to assess negative emotional states in adults. In this project, we developed the Depression Anxiety Stress Scales for Youth (DASS-Y) and tested its psychometric properties. Data were collected from 2,121 Australian children and adolescents aged 7–18 (61% female). This sample was split randomly into a calibration group (*n* = 1075, 61% female) and a cross-validation group (*n* = 1046, 60% female). First, we used Confirmatory Factor Analysis on the calibration group to test the 3-factor DASS model on 40 items we had developed in previous exploratory studies. We then selected the best-performing 21 items based on both statistical and theoretical considerations, guided by the structure and item content of the adult DASS. We cross-validated this new 21-item model in the second half of the sample. Results indicated good fit for the final 21-item 3-factor DASS model in both groups of children and adolescents. Multiple regression analyses showed that when scores on the other DASS-Y scales were held constant, the DASS-Y Depression scale had a strong negative relationship with positive affect and life satisfaction, the DASS-Y Anxiety scale was strongly associated with physiological hyperarousal, and the DASS-Y Stress scale was associated with excessive worrying. However, the relationship between Stress and worrying was only evident from age 10 onwards. Our results show that the core symptoms that define depression, anxiety and stress in children and adolescents are similar to those previously found in adults. The DASS-Y is a public domain instrument that we hope will prove useful in both research and clinical contexts.

## Introduction

Anxiety and mood disorders are among the most prevalent psychiatric disorders in children and adolescents (Kessler et al., 2010; Lawrence et al., 2015). They often persist into adulthood and increase young people’s risk for other mental health problems (de Girolamo et al., 2012). To increase the success of prevention and intervention programs and target treatment to the focus of affective disturbance, a valid and reliable measure of depression and anxiety in childhood and adolescence is crucial. However, existing measures (e.g., Spielberger et al., 1970; Kovacs, 1985) have been shown to assess a mixture of symptoms of both affective states as well as symptoms of general distress, and thereby have limited capacity to discriminate between depression and anxiety (Joiner et al., 1996). Attempts to compensate for these limitations have taken two different directions. Some researchers (Spence, 1998; Chorpita et al., 2000b) have constructed self-report scales to assess dimensions of anxiety and mood disorder symptoms as defined by the Diagnostic and Statistical Manual of Mental Disorders (DSM; American Psychiatric Association [APA], 2000) at the time they carried out their research. However, measures that assess DSM-based diagnostic symptoms, rather than symptoms of pure emotional states of anxiety or depression, are inevitably influenced by the limitations of DSM-defined diagnostic categories for youth (Schniering et al., 2000). Other researchers (Laurent et al., 1999, 2004; Chorpita et al., 2000a) have instead developed new measures that aim to assess pure emotional states in youth by applying Clark and Watson’s (1991) empirically derived tripartite model of negative affect as a guide to item selection.

In their seminal work in adult samples, Clark and Watson (1991) suggested that anxiety and depression share certain characteristics with each other, but that they also have specific symptoms, unique to each. They argued that to improve discrimination between anxiety and depression, assessment instruments should emphasize their specific symptoms and de-emphasize their common characteristics. Subsequently, Watson et al.’s (1995a,b) factor analytic studies suggested that such symptoms as irritability, sad or anxious mood, difficulty concentrating, and sleep and appetite disturbances reflect a general factor of psychological distress, common to both anxiety and depression. They labeled this constellation of symptoms as Negative Affect (NA). They identified a lack of Positive Affect (PA; i.e., a lack of pleasure) as specific to depression, and symptoms of Physiological Hyperarousal (PH; e.g., racing heart, sweaty palms, and dry mouth) as specific to anxiety. Questionnaires within this tripartite framework of NA, PA, and PH were developed for adults (Watson et al., 1995a; Joiner et al., 1999), and were later used to guide the development of similar measures for children and adolescents. The Physiological Hyperarousal Scale for Children (PH-C; Laurent et al., 2004) and the Positive and Negative Affect Schedule for Children (PANAS–C; Laurent et al., 1999) have become widely used youth self-report measures within this framework.

An independent line of research in adults by Lovibond and Lovibond (1995b) came to a similar conclusion regarding many symptoms, such as sleep and appetite disturbance, that have traditionally been thought to assess depression or anxiety but in fact fail to discriminate between them. Their work also supported Clark and Watson’s (1991) emphasis on the importance of autonomic arousal symptoms in the assessment of anxiety, and of anhedonia in the assessment of depression. However, Lovibond and Lovibond (1995b) identified a broader range of core symptoms of anxiety and depression. They found that the specific symptoms of anxiety include a subjective awareness of anxious affect and escape or avoidance tendencies in addition to physiological hyperarousal, and that the specific symptoms of depression include hopelessness, devaluation of life, and self-deprecation in addition to anhedonia. Importantly, Lovibond and Lovibond’s (1995b) research also revealed a third factor that emerged empirically via an aggregation of items reflecting difficulty relaxing, tension, impatience, irritability, and agitation. They labeled this factor ‘tension/stress,’ or ‘stress’ for short. Subsequent research has provided evidence that the ‘stress’ factor reflects a specific emotional syndrome, distinguishable from general distress as well as from anxiety or depression (Lovibond, 1998; Henry and Crawford, 2005). Higher stress scores have been found to be specifically associated with excessive worrying (Huang et al., 2009; Szabó, 2011) and with a diagnosis of generalized anxiety disorder (Brown et al., 1997) in adults.

Based on their findings, Lovibond and Lovibond (1995b) developed a self-report questionnaire, the Depression Anxiety Stress Scales (DASS), designed to assess the full range of core symptoms of each of these emotional states while providing maximal differentiation between them by excluding non-discriminating symptoms. There is considerable published evidence supporting the psychometric properties of the DASS and its brief 21-item version (DASS-21) in adults. The DASS factor structure is stable in clinical and non-clinical samples (Brown et al., 1997; Antony et al., 1998; Clara et al., 2001; Crawford and Henry, 2003; Taylor et al., 2005; Page et al., 2007), as well as in different ethnicities (e.g., Norton, 2007), and across time (Lovibond, 1998). There is evidence of convergent validity with other measures of negative affect (Lovibond and Lovibond, 1995a) and with diagnostic assessments (Brown et al., 1997; Antony et al., 1998). Mean scores are sensitive to intervention, and the DASS has been found to be a valid instrument for routine clinical use (Ng et al., 2007).

In spite of the excellent psychometric properties of the adult DASS and its wide use internationally in research and in clinical practice, there is currently no self-report questionnaire similar to the DASS that is suitable for children and adolescents. Such an instrument would fill an important need by complementing available measures of affect for youth (e.g., Laurent et al., 1999, 2004; Chorpita et al., 2000a) and by providing a comprehensive assessment of all three negative affective states of depression, anxiety, and stress in one brief instrument. At present there is limited evidence as to whether the 3-factor structure of the adult DASS is generalizable to children and adolescents. In addition to the limited evidence regarding the symptoms that characterize anxiety and depression in youth, it is not yet known whether the construct of tension/stress reflected in the adult DASS Stress scale is experienced by children and adolescents (Tully et al., 2009; Patrick et al., 2010; Szabó, 2010). The importance of answering these questions is underlined by the previously mentioned association between the DASS Stress scale and excessive worry and GAD in adults (Brown et al., 1997; Huang et al., 2009; Szabó, 2011). Although excessive worry and GAD (American Psychiatric Association [APA], 2013) represent significant early risk factors toward other psychopathologies (Kertz and Woodruff-Borden, 2011), our knowledge about worrying and its emotional correlates in children and adolescents remains limited (Cartwright-Hatton, 2006; Songco et al., 2020). Earlier theories suggested that children’s worry process is similar to that described in adults by around age 7–8 (Vasey et al., 1994), but more recent evidence indicates that an adult-like worry process is likely to emerge sometime in late childhood or early adolescence (Szabó, 2009, 2007; Carr and Szabó, 2015; Songco et al., 2020). Accordingly, the tension/stress syndrome may also become associated with worrying at those developmental stages, but research showing such an association is lacking.

Therefore, we embarked on a project to further our understanding of negative affect in youth, and to develop a youth version of the DASS (Lovibond and Lovibond, 1995b) based on the observed symptom structure in children and adolescents. In the first stage of this project (Szabó and Lovibond, 2006), we designed a draft list of 76 items aiming to assess the symptoms of depression, anxiety, stress and general distress in age-appropriate language, and tested these in a large group of 7–14-year-old primary school children. A series of exploratory factor analyses showed that, as in adults, such symptoms as fatigue, indecision, appetite or sleep changes or social difficulties reflect general distress and have no strong specific association with either anxiety or depression. Therefore, any measure of specific negative affective states aiming to maximize discriminant power should de-emphasize such symptoms in youth, as in adults. Furthermore, results showed that the core symptoms of depression included devaluation of life, hopelessness, and low self-worth, indicating that depression is defined by a broader range of symptoms than anhedonia and low Positive Affect alone (c.f. Watson et al., 1995b) in children and young adolescents, just as in adults. However, in that study we were unable to differentiate the anxiety and tension/stress constructs. Instead of a 3-factor model of Depression, Anxiety and Stress, the best-fitting model had two factors, one corresponding to Depression and one to a combination of Anxiety and Stress. It is possible, therefore, that tension/stress has not yet emerged as an independent emotional syndrome in 7–14-year-old children. Alternatively, the results may have been influenced by the limitations of the draft items we developed for that study.

Next, guided by these findings (Szabó and Lovibond, 2006), we carried out a series of pilot studies to explore additional items in several independent samples of children and adolescents. These studies tested various draft item sets aiming to assess the symptom structure of negative affect as defined by the adult DASS (Lovibond and Lovibond, 1995b), as well as items that were not included in the adult DASS but were suggested by our adolescent research participants to reflect depression, anxiety or stress (e.g., *“I was stressing about lots of things*,” and “*People told me to chill out”*). Building on these pilot studies, Fowler and Szabó (2013) created 36 new draft items and tested these in 340 12–18-year-old adolescents. Exploratory factor analyses revealed a three-factor structure similar to the adult version of the DASS, although on each scale only 4–6 items had clear and unique loadings on their hypothesized factors. Scores on the stress factor had a specific association with a measure of worry, over and above its association with anxiety and depression. Thus, preliminary evidence suggests that in 12–18-year-old adolescents, the symptom structure of depression, anxiety and stress is present, and that stress has a unique association with worrying, similar to that found in adults (e.g., Huang et al., 2009; Szabó, 2011). However, it is not yet known whether a similar structure would be found in children as well, and whether further items could be identified with clear loadings on their hypothesized factors.

The current report describes the final stage of our substantial multi-part project. We aimed to develop a final 21-item youth version of the DASS (DASS-Y), applicable to both children and adolescents. Guided by our previous results, we tested 40 draft items in a large group of children and adolescents and cross-validated the results in an independent group, using confirmatory factor analyses. We hypothesized that a correlated 3-factor DASS structure reflecting depression, anxiety, and stress would be observable both in children and adolescents. Further, we expected that multiple regression analyses controlling for the intercorrelations among the DASS-Y scales would reveal strong negative relationships between DASS-Y Depression and measures of positive affect and life satisfaction, and a strong positive relationship between DASS-Y Anxiety and a measure of physiological hyperarousal. Concerning DASS-Y Stress, we expected an association with a measure of worrying in adolescents but did not have a specific hypothesis regarding this association in children. Finally, we hypothesized that all three DASS-Y scales would exhibit similar positive associations with a measure of general negative affect.

## Materials and Methods

### Participants

A group of 2121 7–18-year-old Australian children and adolescents took part this study. This sample size was obtained after deleting data from 6 participants whose age was 19 years or older, and from 56 participants with more than 20 percent of missing data from the DASS-Y. The sample included 1258 (61%) females, 829 (39%) males, and 7 participants who did not provide gender information. The mean age of the total sample was 13.04 years (*SD* = 2.76, range = 7.14–18.75 years). Students were recruited from 32 different primary schools (grades 3–6, *n* = 827) and high schools (grades 7–12, *n* = 1294) in Sydney and regional towns. Students were recruited from both public (government) and private (independent or church-affiliated) schools. The socioeconomic characteristics of the sample presented in Table 1 show that the majority of the students lived in areas characterized by high socioeconomic circumstances, were born in Australia, and speak English as the primary language at home.

**TABLE 1:** Distribution of sociodemographic characteristics in the sample.

	Total sample		Primary school		High school	
	*n*	*%*	*n*	*%*	*n*	*%*
**School system**						
Public	903	42.8	674	82.7	229	19.7
Private	1206	57.2	141	17.3	1065	80.3
Total	2109	100.0	815	100.0	1294	100.0
**SES area**						
Low (1–5)	411	20.0	343	43.2	68	5.4
Med (6–8)	526	25.7	156	19.6	370	29.5
High (9–10)	1112	54.3	295	37.2	817	65.1
Total	2049	100.0	794	100.0	1255	100.0
**Country of birth**						
Australia	1885	89.3	719	87.8	1166	90.2
Other	227	10.7	100	12.2	127	9.8
Total	2112	100.0	819	100	1293	100
**Primary language**						
English	1847	91.8	612	83.8	1235	96.3
Other	165	8.2	118	16.2	47	3.7
Total	2012	100.0	730	100.0	1282	100.0
**Other language**						
Yes	692	32.5	520	62.9	172	13.3
No	1429	67.5	307	37.1	1122	86.7
Total	2116	100	827	100	1294	100

*Total n’s are different for each variable due to missing data.*

Nevertheless, a diversity of ethnic backgrounds was reflected in the range of second languages spoken, with 692 (33%) participants reporting that they speak at least one language other than English at home, specifying a total of 51 different languages. The most commonly reported languages were Chinese (including Cantonese, Mandarin, Wu and Teochew, *n* = 223, 10.5% of total sample, 50.2% female), Vietnamese (*n* = 208, 9.8% of total sample, 51.4% female), and Arabic (*n* = 30, 1.5% of total sample, 56.7% female). These language groups were not evenly spread in the sample; the majority of Chinese (82%), Vietnamese (97%) and Arabic (73%) speakers were primary school students. Compared to high school students, primary school students were more likely to speak a second language in addition to English at home [χ*^2^*(1) = 564.36, *p* < 0.001], to live in areas of lower SES [χ*^2^*(2) = 434.37, *p* < 0.001] and to attend public rather than private schools [χ*^2^*(1) = 862.96, *p* < 0.001].

### Procedure

The study procedures were approved by the relevant institutional ethics committees (HREC approval #13545, SERAP approval #2012018) and the principals of each school. We aimed to collect data from 200 students from each grade, with an equal number of genders in each grade and a balanced representation of sociodemographic and ethnic backgrounds across the sample. As shown in Table 1, this aim was only partially achieved, due to practical constraints and difficulties in obtaining school approvals from government schools. In schools where approval was granted, written information and consent forms for parents and participants were sent home with all students by the school, and only students with signed parental and participant consent on the day of testing took part in the study. The only inclusion criterion was attendance in school grades 3–12. Questionnaires were completed on school premises in pen-and-paper written format. Following distribution of the questionnaires, research assistants read a standardized set of instructions asking the participants to read each item and select the most appropriate answer *“to tell us how you feel.”* Participants were reminded that the procedure was entirely voluntary, their responses were confidential, and there were no right or wrong answers. Sociodemographic questions were completed first and the DASS-Y second, followed by the other questionnaires in random order. All students completed the 40 DASS-Y items, other questionnaires were completed by subgroups only to reduce participant workload.

### Materials

#### Background Sociodemographic Information

We collected data about participants’ age in years and months, gender, school system (public or private) and grade, and country of birth. Area of residence was used as a broad proxy for socio-economic status. Areas of residence were coded according to the Australian Bureau of Statistics (Australian Bureau of Statistics [ABS], 2016). Index of Relative Socio-Economic Advantage and Disadvantage (IRSAD), where a score of 1 indicates the lowest (most disadvantaged) 10% of areas, and a decile score of 10 indicates the highest 10%. We combined the IRSAD deciles into three groups representing high (deciles 9 and 10) medium (deciles 6–8) and low (deciles 1–5) Socio-Economic Status (SES) for the purposes of our analyses.

#### Self-Report Questionnaires

##### The Depression Anxiety Stress Scales for Youth

This scale consisted of an initial set of 40 draft items. We tested these 40 items with a view to selecting the best-performing items to create a briefer instrument to match the 21-item adult DASS. Development of the initial 40-item set was informed by the results of previous published (Szabó and Lovibond, 2006; Szabó, 2010; Fowler and Szabó, 2013) and unpublished studies from our research group. In selecting items for inclusion in the current study, we aimed to cover the whole item content of the adult DASS (Lovibond and Lovibond, 1995b). For example, to assess Depression, we aimed to include at least one item to assess each of the ‘Anhedonia’ ‘Dysphoria,’ ‘Hopelessness,’ ‘Devaluation of life,’ ‘Self-deprecation,’ ‘Lack of interest,’ and ‘Inertia’ aspects of this emotional syndrome. However, in our previous studies some items consistently failed to load on their expected factor, even though they had been tested using various wordings in different samples. For example, items attempting to assess inertia consistently failed to load on the depression factor in our previous analyses. Since inertia tended to be relatively weakly associated with depression in adult samples as well, we opted to omit this aspect of depression from the present study. Similarly, the stress item aiming to assess ‘nervous energy’ failed to be consistently associated with other stress items in our previous studies and was not included in the current study. Therefore, not all items originally included in the adult DASS were tested in the current study. We included 13 draft items to assess depression, 14 items to assess anxiety, and 13 items to assess stress. These 40 items are presented in Table 2, which also includes the corresponding items from the adult DASS (Lovibond and Lovibond, 1995b). To provide continuity with its adult counterpart, participants were asked to respond to the DASS-Y items with respect to how they felt during the previous week, using a 4-point Likert-type scale ranging from 0 = “not true” to 3 = “very true.”

**TABLE 2:** The 40 draft DASS-Y items and their adult DASS counterparts.

DEPRESSION	
**DRAFT DASS-Y items**	**ADULT DASS SCALE**

**Anhedonia:**	
Nothing was fun at all.	I couldn’t seem to experience any positive feeling at all.
I did not enjoy anything.	I couldn’t seem to get any enjoyment out of the things I did.
**Dysphoria:**	
I felt down and depressed.	I felt down-hearted and blue.
I could not stop feeling sad.	I felt sad and depressed.
**Hopelessness:**	
There was nothing nice I could look forward to.	I felt that I had nothing to look forward to.
It seemed like nothing would ever work out for me.	I could see nothing in the future to be hopeful about.
**Devaluation of life:**	
I hated my life.	I felt that life was meaningless.
I felt that life was terrible.	I felt that life wasn’t worthwhile.
**Self-deprecation:**	
I felt worthless.	I felt I was pretty worthless.
I felt like I was no good.	I felt I wasn’t worth much as a person.
I hated myself.	
**Lack of interest/involvement:**	
I did not feel excited about anything.	I was unable to become enthusiastic about anything.
I did not feel like doing anything, not even the things I used to enjoy.	I felt that I had lost interest in just about everything.
**Inertia:**	
–	I just couldn’t seem to get going.
–	I found it difficult to work up the initiative to do things.

**ANXIETY**	

**DASS-Y DRAFT items**	**ADULT DASS SCALE**

**Autonomic arousal:**	
I could feel my heart beating really fast, even though I hadn’t done any hard exercise.	I was aware of the action of my heart in the absence of physical exertion (e.g., sense of heart rate increase, heart missing a beat).
My hands got sweaty.	I perspired noticeably (e.g., hands sweaty) in the absence of high temperatures or physical exertion.
My mouth felt dry.	I was aware of dryness of my mouth.
I had trouble breathing (e.g., fast breathing), even when I wasn’t exercising and I was not sick.	I experienced breathing difficulty (e.g., excessively rapid breathing, breathlessness in the absence of physical exertion).
I felt like there was a lump in my throat.	I had difficulty in swallowing.
**Skeletal musculature effects:**	
My hands felt shaky.	I had a feeling of shakiness (e.g., legs going to give way).
–	I experienced trembling (e.g., in the hands).
**Situational anxiety:**	
I was afraid of making a fool of myself.	I was worried about situations in which I might panic and make a fool of myself.
Some situations made me feel so scared that I was relieved when they ended.	I found myself in situations which made me so anxious I was most relieved when they ended.
I felt so nervous that I just wanted to run away.	I feared that I would be “thrown” by some trivial but unfamiliar task.
**Subjective experience of anxious affect:**	
I felt like I was about to panic.	I felt I was close to panic.
I felt terrified.	I felt terrified.
I felt scared for no good reason.	I felt scared without any good reason.
I felt dizzy, like I was about to faint.	I had a feeling of faintness
I was secretly afraid.	

**STRESS**	

**DASS-Y DRAFT items**	**ADULT DASS SCALE**

**Difficulty relaxing:**	
I couldn’t calm down once I was stressed.	I found it hard to calm down after something upset me.
I found it difficult to relax.	I found it difficult to relax.
I could not stop thinking about all the things I had to do.	I found it hard to wind down.
**Easily upset/agitated:**	
I got upset easily.	I found myself getting upset rather easily.
I got upset about little things.	I found myself getting upset by quite trivial things.
I was stressing about lots of things.	I found myself getting agitated.
**Nervous arousal:**	
–	I felt that I was using a lot of nervous energy.
I felt tense and uptight.	I was in a state of nervous tension.
**Irritable/over-reactive:**	
I found myself over-reacting to situations.	I tended to over-react to situations.
I was easily irritated.	I found that I was very irritable.
I was easily annoyed.	I felt that I was rather touchy.
**Impatience**	
I hated it when I had to stop doing what I was doing.	I was intolerant of anything that kept me from getting on with what I was doing.
I found it frustrating when I had to wait for anything (e.g., for a lift or a bus).	I found myself getting impatient when I was delayed in any way (e.g., lifts, traffic lights, being kept waiting).
I got annoyed when people interrupted me.	I found it difficult to tolerate interruptions to what I was doing.

##### The Positive and Negative Affect Schedule for Children – Short Form (PANAS-10; Ebesutani et al., 2012)

The PANAS-10 is a short form of the Positive and Negative Affect Schedule for Children (Laurent et al., 1999), which itself was guided by the adult version (PANAS; Watson et al., 1988). Item selection for the PANAS-C was based on both psychometric and theoretical grounds. The resulting Positive Affect (PA) and Negative Affect (NA) scales demonstrated good convergent and discriminant relationships with existing child self-report measures (Laurent et al., 1999). The PANAS–C asks children to indicate how often they have felt each emotion during the past few weeks on a 5-point Likert-type scale (from 1 = very slightly or not at all to 5 = extremely). The short form PANAS-10 includes 5 items each to assess Negative and Positive affect; therefore, possible total scores ranged from 10 to 50 on the PA and the NA scales. Internal consistency (Cronbach’s alpha) was 0.86 for the Positive Affect scale and 0.84 for the Negative Affect Scale in this study.

##### The Physiological Hyperarousal Scale for Children (PH-C; Laurent et al., 2004)

The PH-C consists of 18-items that assess physiological hyperarousal, defined by the authors as “bodily manifestations of autonomic arousal” (p. 374), and was developed guided by its adult counterpart (Joiner et al., 1999). The PH-C and PANAS-C are often used together to assess tripartite model constructs in youth. Although the PH–C reflects primarily the physical symptoms of panic, symptoms of restlessness and muscle tension usually associated with generalized anxiety disorder are also included. Children are asked to rate on a 5-point scale (1 = very slightly or not at all to 5 = extremely) how often they have experienced symptoms such as “sweaty hands/palms,” or “heart pounding,” during the past 2 weeks. Possible total scores range from 18 to 90. Internal consistency of the PH-C was 0.91 in the current study.

##### The Penn State Worry Questionnaire for Children (PSWQ-C; Chorpita et al., 1997)

The PSWQ-C is an adaptation of the adult Penn State Worry Questionnaire (PSWQ; Meyer et al., 1990). It is a reliable and valid 14-item self-report questionnaire used to assess excessive and uncontrollable worrying in 8–18-year-old children and adolescents (Muris et al., 2001). Participants respond to the items on the PSWQ-C using a 5-point Likert-type scale from 1 = never to 5 = always. In the current sample, internal consistency of the PSWQ-C was 0.93.

##### The Student Life Satisfaction Scale (SLSS; Huebner, 1991a)

This brief 7-item scale assesses the cognitive, evaluative aspects of subjective life satisfaction in children and adolescents, without referring to its emotional aspects (Huebner, 1991b). The SLSS includes such items as “My life is going well” and “I wish I had a different kind of life” (reverse scored). Participants respond to the items with respect to their thoughts about life during the past several weeks. Although the original SLSS has a six-point response format, in the current study response options ranged from 0 = strongly disagree to 3 = strongly agree, to retain consistency with the other measures. Possible total scores ranged from 0 to 21, with higher scores indicating higher life satisfaction. Internal consistency for the SLSS in the current study was 0.84.

Other measures were also included in the questionnaire battery, completed only by subgroups of students. These measures were used to test additional hypotheses unrelated to our current research questions. Therefore, they will not be discussed in the present report.

## Results

### Data Preparation

The data were screened for missing values, normality, and outliers, following the procedures suggested by Byrne (2016). After data were deleted from participants who had more than 20% missing DASS-Y items, all other missing values were replaced using the Maximum Likelihood (ML) estimation procedure in SPSS-24. All variables were found to be non-normally distributed, but there were only 4 items with skew above 2 (largest skew was 2.48), and there were no variables with kurtosis above 7 (largest kurtosis = 5.58). Mahalanobis distance calculations were used to identify multivariate outliers. Data from outliers were retained, as repeating the analyses with and without these participants did not reveal a substantial effect on the pattern of results.

### Confirmatory Factor Analyses: Developing and Testing the 21-Item Depression Anxiety Stress Scales for Youth

#### Data Analysis Plan

For the purposes of the factor analyses, the sample was randomly split into two approximately equal groups. Group 1 (*N* = 1075, 60.8% female, *M*_age_ = 12.95, *SD* = 2.75) was used as a calibration sample to test the 40 draft items and construct an improved 21-item model. Group 2 (*N* = 1046, 60.3% female, *M*_age_ = 13.13, *SD* = 2.79 years) was used as a cross-validation sample to test the new 21-item model in an independent group of participants. Following the factor analyses, the total sample was used (*N* = 2121, see Table 1) to provide descriptive statistics of the new DASS-Y, and to test our predictions concerning its relationships with other measures.

Confirmatory Factor Analyses (CFA) with Maximum Likelihood (ML) estimation using AMOS-24 were conducted (see Olsson et al., 2000; Brown, 2015; Byrne, 2016). Model fit was evaluated with respect to a variety of fit indices. To complement the commonly used *Chi-square* statistic indicating the discrepancy between the hypothesized model and that implied by the data, we examined the *Comparative Fit Index* (*CFI*), the Tucker-Lewis Index (*TLI*), the *Root Mean Square Error of Approximation* (*RMSEA*) and its 90% confidence interval, and the *standardized Root Mean Square Residual* (*SRMR*). For the *CFI and TLI*, values above 0.90 are generally accepted as suggesting an acceptable fit to the data, and a cut-off of 0.95 indicates good fit. For the RMSEA and SRMR, smaller values indicate better fit. RMSEA values below 0.08 have been suggested as indicating acceptable fit and those below 0.06 excellent fit. SRMR values below 0.05 indicate excellent fit (see Brown, 2015; Byrne, 2016).

#### Group 1: Initial Confirmatory Factor Analyses of the 40 Draft Items

An initial 40-item three-factor confirmatory model was constructed, with items allocated to their respective depression, anxiety and stress factors as described in Table 2 in the Section “Materials and Methods.” The three factors were allowed to correlate. This model was tested separately in primary school (grades 3–6) and in high school (grades 7–12) students. The model fit indices presented in Table 3 indicated that this initial model provided a reasonable but not a good fit to the data. The correlation matrix among all 40 draft items is provided in Supplementary Table 1. Factor loadings for the model including all 40 draft items separately for primary school and high school students are in Supplementary Table 2.

**TABLE 3:** Model fit indices for the 40-item draft and the 21-item final DASS-Y models in the calibration sample (*N* = 1075).

		Model fit indices						
	*n*	χ^2^	DF	CFI	TLI	RMSEA	RMSEA 90% C.I.	SRMR
**40-item draft DASS-Y**								
Primary school	435	2021.4	737	0.80	0.80	0.063	0.060, 0.067	0.058
High school	640	3470.3	737	0.84	0.83	0.076	0.074, 0.079	0.058
**21-item DASS-Y**								
Primary school	435	479.1	186	0.90	0.89	0.060	0.054, 0.067	0.055
High school	640	972.8	186	0.91	0.89	0.081	0.076, 0.086	0.055
**21-item DASS-Y with correlated error**								
Primary school	435	368.9	181	0.94	0.93	0.049	0.042, 0.056	0.046
High school	640	637.2	181	0.95	0.94	0.063	0.058, 0.068	0.043
Total group	1075	678.7	181	0.95	0.95	0.051	0.047, 0.055	0.036

*CFI, Comparative Fit Index; TLI, Tucker–Lewis Index; RMSEA, Root Mean Square Error of Approximation; RMSEA 90% C. I., 90% confidence interval for the RMSEA; SRMR, Standardized Root Mean Square Residual.*

##### Development of a Revised Model

Consistent with our research plan, we examined whether model fit could be improved by removing poorly performing items. We aimed to create a shortened 21-item version of the DASS-Y that would achieve maximal discrimination between the three factors, maximal coverage of the whole symptom content of each affective state, and applicability to the whole age range. Thus, in selecting items for removal, we took into account both empirical and theoretical considerations. First, we examined the size of individual item loadings and flagged items with the lowest loadings for potential removal. Next, we examined modification indices for regression weights (factor loadings), and flagged items for removal if there was a suggestion of item misspecification or cross-loading. Finally, we considered the results of our previous studies indicating whether previous versions of flagged items had also performed poorly. In addition to these empirical considerations, we aimed to retain as much coverage as possible of the various aspects of each affective state, as specified by the DASS model (see Table 2).

For example, the item *“I did not feel excited about anything”* was removed from the depression scale because it was consistently the lowest loading item on Depression in both the primary school and high school groups, and the same or similar items also had weak performance in our previous studies. Although the item *“I felt down and depressed”* received relatively higher loadings, it was also removed from the depression scale because modification indices for this item suggested that it cross-loads onto anxiety or stress in both primary and high school students. In addition, similar items with alternative wordings (for example, “*I felt sad*”) repeatedly failed to show strong specific relationships with depression in our previous studies (Szabó and Lovibond, 2006; Fowler and Szabó, 2013). Other items found to cross load to anxiety or stress (e.g., *I did not feel like doing anything*…) in the present and in previous studies were also removed from the depression scale. The same considerations directed the removal of other items, for example the item “*I felt tense and uptight*” from the Stress scale, and “*I was afraid of making a fool of myself*” from the Anxiety scale. Following tests of several successive models, we retained 7 items for each of the three DASS-Y scales. Model fit indices for this 21-item DASS-Y model are included in Table 3. Although model fit indices were in the acceptable range, we examined modification indices for this 21-item model as well, to identify any further misspecifications in the two school groups separately. Modification indices suggested that several item pairs had substantial error covariance. Covariances between error terms were freed up when the modification indices were high and the covariation between items made substantive sense. For example, two item pairs on the depression scale had substantial error covariation in both school groups. In one pair, both items reflected a lack of enjoyment and interest in activities (*“I did not enjoy anything”, “I had nothing to look forward to”*). In the other pair, both items reflected negative evaluations of life in general (*“I hated my life,” “I felt that life was terrible”*). There were three similar item pairs on the Stress factor. Therefore, we allowed 5 error covariances to be freely estimated in the final model. Model fit indices for this final model indicated excellent fit in the high school and primary school groups, as shown in Table 3.

**FIGURE 1:**
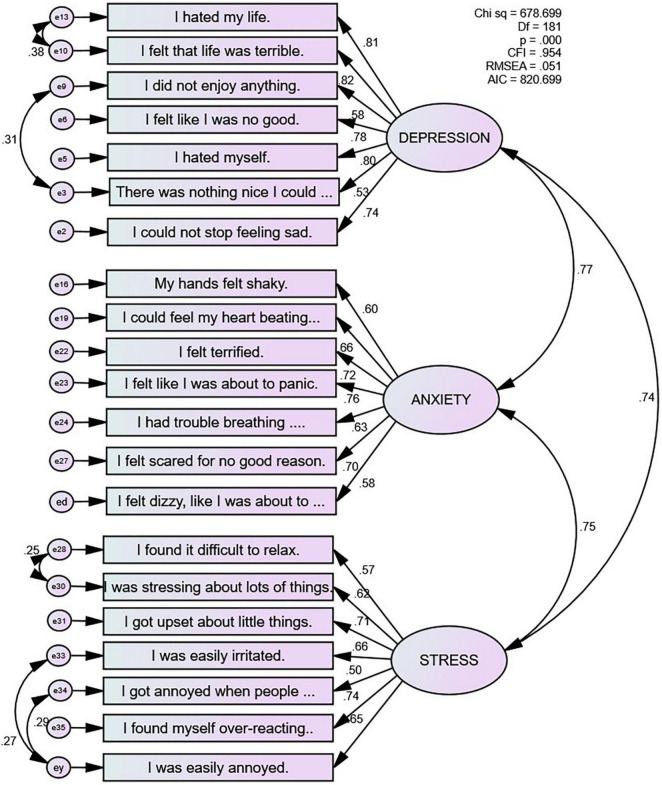
The 21-item DASS-Y model in the calibration sample (*N* = 1075).

All factor loadings were significant in the primary and high school groups. Model fit indices from an unconstrained multigroup analysis indicated that the covariance structures were similar between the two school groups (*n* = 1075, *df* = 362, χ^2^ = 1006.07, *CFI* = 0.94, *TLI* = 0.94, *RMSEA* = 0.041, *RMSEA 90% C.I.* = 0.038–0.044, *SRMR* = 0.046). A second multigroup model with factor loadings constrained equal between the two school groups showed that although there was a small, statistically significant drop in chi square (Δχ*^2^* = 31.04, *df* = 18, *p* = 0.028) all other model fit indices remained unchanged (*n* = 1075, *df* = 380, χ^2^ = 1037.11, *CFI* = 0.94, *TLI* = 0.94, *RMSEA* = 0.040, *RMSEA 90% C.I.* = 0.037–0.043, *SRMR* = 0.047) compared to the unconstrained model. Therefore, we combined the primary and high school groups and present the model fit indices for the calibration sample as a whole in the last row of Table 3. Figure 1 illustrates the final 21-item DASS-Y model for the calibration sample as a whole.

#### Group 2: Cross-Validating the 21-Item Depression Anxiety Stress Scales for Youth Model

We next tested the new, 21-item three-factor model with the same 5 correlated error terms in our independent cross-validation sample of 1,046 children and adolescents (Group 2). Model fit indices presented in Table 4 show that the three-factor model we identified in Group 1 also fit the data well in Group 2. Model fit indices from an unconstrained multigroup analysis indicated that the covariance structures were similar between the primary and high school groups (*n* = 1,046, *df* = 362, χ^2^ = 1,129.29, *CFI* = 0.93, *TLI* = 0.92, *RMSEA* = 0.045, *RMSEA 90% C.I.* = 0.042–0.048, *SRMR* = 0.047). A second multigroup model with factor loadings constrained equal between the two school groups showed that although there was a small, statistically significant drop in chi square (Δχ*^2^* = 31.11, *df* = 18, *p* = 0.028), all other model fit indices remained unchanged (*n* = 1,046, *df* = 380, χ^2^ = 1160.40, *CFI* = 0.93, *TLI* = 0.92, *RMSEA* = 0.044, *RMSEA 90% C.I.* = 0.041–0.047, *SRMR* = 0.047) compared to the unconstrained model. Therefore, we combined the two school groups, and we present model fit indices for the cross-validation sample as a whole in the third row of Table 4.

**TABLE 4:** Model fit indices for the 21-item DASS-Y in the cross-validation sample (*N* = 1046), and for multigroup comparisons between the calibration and cross-validation samples.

		Model fit indices						
	*n*	χ^2^	DF	CFI	TLI	RMSEA	RMSEA 90% C.I.	SRMR
Primary school	392	372.7	181	0.93	0.92	0.052	0.045, 0.060	0.047
High school	654	756.6	181	0.93	0.91	0.070	0.065, 0.075	0.048
Total group 2	1046	775.7	181	0.94	0.93	0.056	0.052, 0.060	0.042
Multigroup 1	2121	1454.4	362	0.95	0.94	0.038	0.036, 0.040	0.036
Multigroup 2	2121	1483.4	380	0.95	0.94	0.037	0.035, 0.039	0.038

*CFI, Comparative Fit Index; TLI, Tucker–Lewis Index; RMSEA, Root Mean Square Error of Approximation; RMSEA 90% C. I., 90% confidence interval for the RMSEA; SRMR, Standardized Root Mean Square Residual.*

Finally, we compared model fit between the calibration sample (*n* = 1075) and the cross-validation sample (*n* = 1046). Results for the unconstrained ‘Multigroup 1’ model in Table 4 indicated that the covariance structures were similar between the two independent samples. Compared to this unconstrained model, there was a small, marginally statistically significant change in chi square in the ‘Multigroup 2’ model, where factor loadings were set to be equal between the calibration sample and the cross-validation sample (Δχ*^2^* = 29.00, *df* = 18, *p* = 0.048). All other model fit indices indicated excellent fit for this model as well, as shown in the last row of Table 4. Factor loadings obtained from the cross-validation sample are presented in Table 5.

**TABLE 5:** Factor loadings for the 21-item DASS-Y in the cross-validation sample (*N* = 1046).

	Depression	Anxiety	Stress
I hated my life.	0.833		
I hated myself.	0.803		
I felt that life was terrible.	0.784		
I felt like I was no good.	0.783		
I could not stop feeling sad.	0.686		
I did not enjoy anything.	0.603		
There was nothing nice I could look forward to.	0.539		
I felt like I was about to panic.		0.711	
I felt terrified.		0.682	
I felt scared for no good reason.		0.656	
I could feel my heart beating.		0.632	
I had trouble breathing.		0.620	
My hands felt shaky.		0.584	
I felt dizzy, like I was about to faint.		0.543	
I got upset about little things.			0.745
I was easily irritated.			0.704
I found myself over-reacting to situations.			0.698
I was easily annoyed.			0.687
I was stressing about lots of things.			0.617
I got annoyed when people interrupted…			0.521
I found it difficult to relax.			0.511

#### Relationships Between the 21-Item Depression Anxiety Stress Scales for Youth and Other Measures of Negative Affect

Next, we combined the calibration and cross-validation samples and investigated the correlates of the three DASS-Y scales in primary school and high school students separately. The correlations of the DASS-Y scales with related measures are presented in Table 6. All measures correlated with each other in the expected direction in both primary and high school students.

**TABLE 6:** Correlations between the DASS-Y scales and other measures in primary school and high school students.

	DASS-Y depression		DASS-Y anxiety		DASS-Y stress		PSWQ-C worry		Physiological hyperarousal		PANAS positive affect		PANAS negative affect	
	*r*	*n*	*r*	*n*	*r*	*N*	*r*	*n*	*r*	*n*	*r*	*n*	*r*	*n*
**Primary school**														
**Grades 3–6**														
DASS-Y depression	1.00													
DASS-Y anxiety	0.59**	827	1.00											
DASS-Y stress	0.59**	827	0.58**	827	1.00									
PSWQ-C worry	0.60**	226	0.55**	226	0.66**	226	1.00							
Physiological hyperarousal	0.70**	41	0.80**	41	0.60**	41	0.77**	41	1.00					
PANAS positive affect	−0.48**	90	**−**0.27 *	90	**−**0.33**	90	**−**0.26 *	89	**−**0.27	41	1.00			
PANAS negative affect	0.63**	90	0.67**	90	0.62**	90	0.67**	89	0.66**	41	**−**0.29**	40	1.00	
SLSS life satisfaction	−0.63**	737	**−**0.34**	737	**−**0.38**	737	**−**0.38**	172	**−**0.53**	40	0.41**	40	**−**0.51**	40
**High school**														
**Grades 7–12**														
DASS-Y depression	1.00													
DASS-Y anxiety	0.71**	1294	1.00											
DASS-Y stress	0.63**	1294	0.66**	1294	1.00									
PSWQ-C worry	0.56**	744	0.61**	744	0.70**	744	1.00							
Physiological hyperarousal	0.54**	638	0.79**	638	0.63**	638	0.56**	637	1.00					
PANAS positive affect	−0.54**	722	**−**0.36**	722	**−**0.41**	722	**−**0.43**	721	**−**0.24**	625	1.00			
PANAS negative affect	0.73**	731	0.74**	731	0.68**	731	0.67**	730	0.66**	635	**−**0.39**	719	1.00	
SLSS life satisfaction	−0.66**	1231	**−**0.47**	1231	**−**0.47**	1231	**−**0.48**	707	**−**0.41**	606	0.59**	687	**−**0.56**	696

**Correlation is significant at the 0.01 level (2-tailed). *Correlation is significant at the 0.05 level (2-tailed).

*Values in bold refer to correlations that were predicted to be particularly strong on theoretical grounds.*

*PSWQ-C Worry, The Penn State Worry Questionnaire for Children (Chorpita et al., 1997).*

*Physiological Hyperarousal, The Physiological Hyperarousal Scale for Children (PH-C; Laurent et al., 2004).*

*PANAS Positive Affect, The Positive and Negative Affect Schedule for Children – short form, Positive Affect scale (PANAS-10; Ebesutani et al., 2012).*

*PANAS Negative Affect, The Positive and Negative Affect Schedule for Children – short form, Negative Affect scale (PANAS-10; Ebesutani et al., 2012).*

*SLSS Life satisfaction, The Student Life Satisfaction Scale (Huebner, 1991a).*

To investigate the associations of each DASS-Y scale with measures expected to have a specific relationship with DASS-Y Depression, Anxiety, and Stress, we conducted a series of multiple regression analyses. In these analyses, the three DASS-Y scales were entered simultaneously as predictors to control for their intercorrelations. Table 7 presents the results obtained for the primary school and high school samples, respectively. Results show that DASS-Y Depression had the highest association with PANAS Positive Affect and with Life satisfaction scores, DASS-Y Anxiety was highly positively associated with Physiological hyperarousal, and DASS-Y Stress was strongly associated with PSWQ-C scores. Conversely, all three DASS-Y scales had a similar strength of association with PANAS negative affect.

**TABLE 7:** DASS-Y scales predicting scores on measures of positive affect, life satisfaction, physiological hyperarousal, excessive worry, and negative affect in the primary and high school samples separately.

Primary school sample						
**Predictors**	***R* ^2^**	***F***		***Beta***	***t***	
**Dependent variable: PANAS-PA (*n* = 90)**						
	0.23	8.65	****			
DASS-Y depression				−0.45	−3.55	***
DASS-Y anxiety				0.04	0.33	
DASS-Y stress				−0.09	−0.69	
**Dependent variable: SLSS (*n* = 737)**						
	0.40	162.28	****			
DASS-Y depression				−0.64	−16.78	****
DASS-Y anxiety				0.05	1.43	
DASS-Y stress				−0.03	−0.86	
**Dependent variable: PH-C (*n* = 41)**						
	0.73	33.13	****			
DASS-Y depression				0.32	2.82	**
DASS-Y anxiety				0.56	4.94	****
DASS-Y stress				0.09	0.77	
**Dependent variable: PSWQ-C (*n* = 226)**						
	0.52	80.62	****			
DASS-Y depression				0.26	4.24	****
DASS-Y anxiety				0.15	2.50	*
DASS-Y stress				0.42	6.78	****
**Dependent variable: PANAS-NA (*n* = 88)**						
	0.57	36.51	****			
DASS-Y depression				0.26	2.70	**
DASS-Y anxiety				0.37	3.89	****
DASS-Y stress				0.25	2.63	**

**High school sample**						

**Predictors**	***R* ^2^**	***F***		***Beta***	***t***	

**Dependent variable: PANAS-PA (*n* = 722)**						
	0.30	103.60	****			
DASS-Y depression				−0.51	−10.98	****
DASS-Y anxiety				0.11	2.20	*
DASS-Y stress				−0.17	3.82	****
**Dependent variable: SLSS (*n* = 1231)**						
	0.44	323.84	****			
DASS-Y depression				−0.62	19.60	****
DASS-Y anxiety				0.04	1.17	
DASS-Y stress				−0.11	3.72	****
**Dependent variable: PH-C (*n* = 638)**						
	0.65	400.11	****			
DASS-Y depression				−0.11	−3.15	***
DASS-Y anxiety				0.73	20.41	****
DASS-Y stress				0.21	6.29	****
**Dependent variable: PSWQ-C (*n* = 744)**						
	0.53	284.25	****			
DASS-Y depression				0.10	2.85	**
DASS-Y anxiety				0.20	5.20	****
DASS-Y stress				0.50	14.32	****

**Primary school sample**						

**Dependent variable: PANAS-NA (*n* = 731)**						
	0.66	479.97	***			
DASS-Y depression				0.35	11.02	****
DASS-Y anxiety				0.32	9.74	****
DASS-Y stress				0.25	8.29	****

*p < 0.05, **p < 0.01, ***p < 0.005, ****p < 0.001.

*PANAS-PA, The Positive and Negative Affect Schedule for Children – short form, Positive Affect scale (PANAS-10; Ebesutani et al., 2012).*

*SLSS, The Student Life Satisfaction Scale (Huebner, 1991a).*

*PH-C, The Physiological Hyperarousal Scale for Children (Laurent et al., 2004).*

*PSWQ-C, The Penn State Worry Questionnaire for Children (Chorpita et al., 1997).*

*PANAS-NA, The Positive and Negative Affect Schedule for Children – short form, Negative Affect scale (PANAS-10; Ebesutani et al., 2012).*

##### The Unique Association Between Stress and Worrying

An important theoretical question we wished to investigate in this study was the emergence of a unique association between stress and worrying. To explore whether this specific association is present across the whole target age group, we conducted multiple regression analyses in each school grade, with PSWQ-C scores as the criterion variable and the three DASS-Y scales entered simultaneously as predictors to control for their intercorrelations. The results in Table 8 show that in Grade 3, PSWQ-C scores had a stronger association with DASS-Y Depression, compared to DASS-Y Stress or DASS-Y Anxiety. In Grade 4, DASS-Y Depression and DASS-Y Stress had similar associations with PSWQ-C scores. A clear relationship of worry with DASS-Y Stress emerged in Grade 5 and remained stable thereafter.

**TABLE 8:** Simultaneous multiple regression analyses predicting total scores on the PSWQ-C in each school grade separately.

Grade	Predictors	*R* ^2^	*F*		*Beta*	*t*	
Grade 3 *n* = 55		0.44	13.38	***			
	DASS-Y depression				0.60	3.97	***
	DASS-Y Aaxiety				−0.02	−0.12	
	DASS-Y stress				0.11	0.76	
Grade 4 *n* = 60		0.49	18.20	***			
	DASS-Y depression				0.32	2.60	**
	DASS-Y anxiety				0.22	1.85	
	DASS-Y stress				0.30	2.27	*
Grade 5 *n* = 90		0.65	52.57	***			
	DASS-Y depression				0.18	2.07	*
	DASS-Y anxiety				0.26	3.03	**
	DASS-Y stress				0.50	6.13	***
Grade 6 *n* = 21		0.59	8.22	***			
	DASS-Y depression				−0.14	−0.70	
	DASS-Y anxiety				0.42	1.92	
	DASS-Y stress				0.52	2.37	*
Grade 7 *n* = 140		0.62	73.11	***			
	DASS-Y depression				0.22	3.19	**
	DASS-Y anxiety				0.22	2.93	*
	DASS-Y stress				0.46	6.23	***
Grade 8 *n* = 191		0.52	67.19	***			
	DASS-Y depression				0.11	1.31	
	DASS-Y anxiety				0.26	3.34	**
	DASS-Y stress				0.43	5.95	***
Grade 9 *n* = 235		0.58	105.87	***			
	DASS-Y depression				0.07	1.11	
	DASS-Y anxiety				0.19	2.76	**
	DASS-Y stress				0.57	9.65	***
Grade 10 *n* = 163		0.40	35.91	***			
	DASS-Y depression				0.05	0.57	
	DASS-Y anxiety				0.18	1.94	
	DASS-Y stress				0.46	5.46	***

*p < 0.05, **p < 0.01, ***p < 0.001.

*Data for PSWQ-C were only available for 11 students in Grade 11 and 3 students in Grade 12, therefore data for Grades 11 and 12 were not included in these analyses.*

*PSWQ-C, The Penn State Worry Questionnaire for Children (Chorpita et al., 1997).*

##### Descriptive Statistics

Reliability estimates were calculated for the whole group (*N* = 2121). Cronbach’s alpha values were α = 0.89 for Depression, α = 0.84 for Anxiety, and α = 0.84 for Stress. McDonald’s omega values were ω = 0.90 for Depression, ω = 0.84 for Anxiety, and ω = 0.84 for Stress.

Table 9 lists mean scores and standard deviations for the DASS-Y. We conducted three 2(gender) × 2(school) univariate Analyses of Variance (ANOVAs) to test differences in mean scores. Results for Depression showed that girls had higher scores than boys overall [*F*(1,2110) = 17.64, *p* < 0.001] and that primary school students reported higher scores than high school students overall [*F*(1,2110) = 25.43, *p* < 0.001]. These main effects were qualified by a significant interaction between gender and school [*F*(1,2110) = 11.96, *p* < 0.001]. For Anxiety, the pattern of significant findings was the same, indicating a main effect of gender [*F*(1,2110) = 24.96, *p* < 0.001] and school [*F*(1,2110) = 33.00, *p* < 0.001], and their interaction [*F*(1,2110) = 16.67, *p* < 0.001]. For Stress, there was a main effect of gender [*F*(1,2110) = 40.18, *p* < 0.001] but not of school [*F*(1,2110) = 1.14, *p* = 0.28]. This finding was also qualified by a significant interaction [*F*(1,2110) = 41.60, *p* < 0.001]. Follow-up tests of simple effects showed that in high school, girls had significantly higher scores than boys for Depression, Anxiety and Stress (all *p*s < 0.001), but that in primary school there were no significant differences between the genders. When comparing scores between primary and high schools, there were no differences in anxiety and depression scores between high school and primary school for girls, but stress scores were higher in high school (*p* < 0.001). Among boys, scores on all three DASS-Y scales were higher in primary school than in high school (all *p*s < 0.001).

**TABLE 9:** DASS-Y descriptive statistics for boys and girls in the total sample and in the two school groups.

		DASS-Y depression		DASS-Y anxiety		DASS-Y stress	
		*M*	*SD*	*M*	*SD*	*M*	*SD*
**Primary school**							
Female	*n* = 432	4.03	4.74	4.02	4.25	7.61	4.82
Male	*n* = 394	3.88	4.45	3.85	3.89	7.64	4.57
Total	*N* = 826	3.96	4.60	3.93	4.08	7.62	4.70
**High school**							
Female	*n* = 853	3.70	5.10	3.70	4.61	9.31	5.34
Male	*n* = 435	2.08	3.65	1.99	3.15	6.41	4.74
Total	*N* = 1288	3.15	4.72	3.13	4.25	8.34	5.10
**Total sample**							
Female	*n* = 1285	3.81	4.98	3.81	4.49	8.74	5.23
Male	*n* = 829	2.93	4.15	2.87	3.64	6.99	4.70
Total	*N* = 2121^a^	3.46	4.69	3.44	4.20	8.05	5.09

*^a^n = 7 missing gender information.*

## Discussion

The present study built upon our previous published and unpublished exploratory data to investigate the symptom structure of negative affect in youth. We developed a new instrument, the Depression Anxiety Stress Scales for Youth (DASS-Y), as a comprehensive measure of negative affect in both children and adolescents. We tested Australian children in grades 3–12 (aged 7–18), consistent with our aim to develop an instrument available for a wide age range. Results of CFAs in independent calibration and cross-validation samples indicated good fit for the final 21-item 3-factor DASS-Y in both children and adolescents.

The Depression factor was defined by items reflecting anhedonia, dysphoria, devaluation of life, self-deprecation, and hopelessness. Devaluation of life and self-deprecation were the strongest indicators of this construct. In contrast, items reflecting a general lack of interest or enthusiasm, symptoms that perform well in the adult DASS model, did not strongly or uniquely contribute to the definition of the depression construct. Such items as *“I did not feel like doing anything*…” did not consistently or strongly load on the depression factor in the current research, as in our previous studies. Although the final version of the DASS-Y Depression scale does not include items reflecting a lack of enthusiasm or excitement, a lack of pleasure in usual activities is reflected in such items as *“I did not enjoy anything,”* consistent with the emphasis on such symptoms as a diagnostic indicator in the DSM-5 (American Psychiatric Association [APA], 2013). The Anxiety factor was defined by items reflecting autonomic arousal, skeletal musculature effects, and a subjective awareness of anxious affect, with a sense of impending panic being the strongest indicator of this construct. Although situational anxiety and escape or avoidance tendencies are important features of anxiety, we decided against retaining items reflecting such symptoms. Two of these items had relatively low factor loadings on Anxiety (e.g., “*making a fool of myself*”). Another item (*“I was so nervous that I wanted to run away”*) had a very strong factor loading on Anxiety, but it also had a strong association with the Depression factor. Because we aimed to retain items that would help us achieve maximal discrimination, we decided to remove this item from the DASS-Y. Finally, the Stress factor was defined by items reflecting a difficulty relaxing and a tendency to be over-reactive, irritable, impatient, and easily upset. With the exception of nervous arousal for which we had no candidate item from our previous studies, we retained the broad symptom structure suggested by the adult DASS model.

Our assessments of the relationships of the DASS-Y scales to related tripartite constructs yielded the predicted pattern of results. As expected, scores on the Depression scale showed moderately strong negative correlations with Positive Affect (Ebesutani et al., 2012), and the Anxiety scale was strongly associated with Physiological Hyperarousal (Laurent et al., 2004). The high correlation between DASS-Y Anxiety and Physiological Hyperarousal scores reflects the fact that DASS-Y Anxiety, much like its adult counterpart, is primarily defined by symptoms of autonomic arousal, although both the adult and the youth versions also include items reflecting a subjective awareness of anxious affect (Lovibond and Lovibond, 1995b). In addition to its association with low positive affect, the DASS-Y Depression scale also had strong positive associations with Life Satisfaction (Huebner, 1991a) scores. This finding is consistent with the fact that within the framework of the DASS model, the unique core symptoms of depression involve a broader range of experiences than low positive affect.

While this pattern of associations regarding depression and anxiety was expected based on previous studies, the viability and correlates of the DASS-Y Stress scale were less clear in advance, especially in the younger age range. However, the results showed a similar symptom profile for this scale in the DASS-Y as in the adult DASS, and a similar pattern of associations with other measures. First, DASS-Y Stress scores were not more strongly associated with the tripartite construct of Negative Affect (NA) than were the other two DASS-Y scales. This finding suggests that, as in adults, DASS-Y Stress is not merely a measure of negative affect. Moreover, excessive worrying as measured by the PSWQ-C (Chorpita et al., 1997) had a relatively stronger association with DASS-Y Stress scores, compared to Depression or Anxiety. Nevertheless, a unique association with worrying was not uniformly present through the whole age range. When we tested the associations between the three DASS-Y scales and worry scores using multiple regression analyses in each school grade separately, we observed that a strong unique association between worry and stress became clear in Grade 5 (mean age 10.7 years) and it remained present thereafter.

This pattern of findings adds another piece of evidence to our understanding of the development of worrying and its functions. Specifically, it has been suggested that an important function of worrying is to switch from imagery to verbal thinking, a process which enables the individual to reduce the aversive physiological symptoms of anxiety (Borkovec et al., 2004). While it has previously been suggested that this emotional avoidance process may be present in children from 8 years of age (Vasey et al., 1994), our results do not clearly support this proposition. Our data suggest that worrying is not uniquely associated with autonomic arousal symptoms at any age. Instead, it may be associated with depression at age 8–9 years, and it is clearly associated with stress from about 10–11 years of age and thereafter. These findings are consistent with the idea that an adult-like worry process does not emerge at age 8 as previously thought (Vasey et al., 1994), but that it becomes more similar to the adult process involving verbal thinking and problem-solving attempts during late childhood or early adolescence (Szabó, 2007, 2009; Carr and Szabó, 2015; Songco et al., 2020).

Descriptive statistics indicated that girls obtain higher scores than boys on all three DASS-Y scales in high school, but there was no difference between the genders in primary school. This pattern is broadly consistent with previous data indicating that gender differences in negative affect and related disorders tend to emerge during adolescence (Hale et al., 2008; Ohannessian et al., 2017; Salk et al., 2017). An unexpected finding in the present study, however, was that the emergence of gender differences in negative emotion appeared to be partly due to a reduction in DASS-Y scores for boys in high school compared to primary school. Due to systematic differences between the school groups, such differences need to be interpreted with caution. As described in the Method section, primary schools included a high percentage of children residing in lower SES areas, those from non-English-speaking backgrounds, and from public schools, and the opposite pattern was observed for high schools. Therefore, any differences in DASS-Y scores between high school and primary school students were likely to be influenced by a complex interaction between these sociodemographic variables and age, rather than by age alone.

### Limitations and Future Research

Due to practical constraints on our sampling, we could not ascertain that different school cohorts were matched on sociodemographic variables. The present samples were collected in order to determine the factor structure of the DASS-Y, and should not be treated as normative data, which would require a formal stratified sampling process to ensure representativeness of the general population. In addition, longitudinal studies are needed to test the reliability of any age-related changes. As previous research examining the prevalence of anxiety disorder symptoms has shown, such changes are complex and include both increases and decreases of specific symptom patterns at different ages (Hale et al., 2008). Future studies also need to examine the properties of the DASS-Y in help-seeking samples. Research with the adult DASS has demonstrated that scale scores are much higher in clinical samples, but the factor structure is essentially identical to that observed in non-clinical samples (e.g., Brown et al., 1997; Antony et al., 1998; Page et al., 2007). Nonetheless it would be valuable to test whether the same is true for the DASS-Y, to check for ceiling effects, and to assess sensitivity to change in symptom severity over the course of treatment. Finally, testing the correlated 3-factor DASS model against other, theoretically possible models of the structure of negative affect in children and adolescents would also be an important avenue for further research.

### Clinical Implications

The ability to reliably measure and distinguish between negative affective states is critical for research that attempts to identify the common and specific aetiological factors and proximal mechanisms that underlie each state. Our research shows that depression, anxiety, and stress can be reliably distinguished in children and adolescents. Better understanding of these states will in turn inform clinical interventions targeted to particular syndromes. While there are undoubtedly common causal factors for depression, anxiety, and stress, which justify *trans*-diagnostic interventions, the differences between these syndromes are also meaningful. To develop syndrome-specific interventions, we need to be able to measure each syndrome as accurately as possible. This was the primary aim of the present research.

### Conclusion

Our results show that it is possible to identify a core set of symptoms that define depression, anxiety and stress in children and adolescents, and that these symptoms are similar to those previously established in adults (Lovibond and Lovibond, 1995a,b). In particular, our findings indicate that a stress construct can also be assessed in youth via self-report, and that it is associated with worrying in children and adolescents from grade 5 (about 10–11 years of age) onwards. Our data suggest that the DASS-Y provides a psychometrically sound brief dimensional measure of depression, anxiety and stress in children and adolescents aged 8–17. The availability of this measure fills an important gap in our current repertoire of instruments for assessing negative emotional states in youth. Like the tripartite model, the DASS-Y is empirically based and provides scores that are graded in severity, reflecting the distribution of symptom levels observed in the population. However, the DASS-Y excludes non-specific indicators of general distress, and it assesses an additional coherent syndrome of stress. The DASS-Y is a public domain instrument, and, like the adult DASS, we believe it will prove useful in both research and clinical contexts.

## Data Availability Statement

The raw data supporting the conclusions of this article will be made available by the authors, without undue reservation, to any qualified researcher.

## Ethics Statement

The studies involving human participants were reviewed and approved by The University of Sydney Human Research Ethics Committee approval #13545. Written informed consent to participate in this study was provided by the participants’ legal guardian/next of kin.

## Author Contributions

MS took primary responsibility for study design, data collection, data analysis, and manuscript preparation. PL contributed to study design, data analysis, and manuscript preparation. Both authors reviewed and accepted this manuscript in its final form.

## Conflict of Interest

The authors declare that the research was conducted in the absence of any commercial or financial relationships that could be construed as a potential conflict of interest.

## Publisher’s Note

All claims expressed in this article are solely those of the authors and do not necessarily represent those of their affiliated organizations, or those of the publisher, the editors and the reviewers. Any product that may be evaluated in this article, or claim that may be made by its manufacturer, is not guaranteed or endorsed by the publisher.
