# Supplementary material for: Development and Psychometric Properties of the DASS-Youth (DASS-Y): An Extension of the Depression Anxiety Stress Scales (DASS) to Adolescents and Children
Source: Front Psychol. 2022 Apr 14;13:766890. doi: 10.3389/fpsyg.2022.766890 (PMC9047499; doi:10.3389/fpsyg.2022.766890)
Supplement: Supplementary file 1 [file Table_1.DOCX]

Supplementary Table 1.

Bivariate correlations among the 40 draft DASS-Y items in the calibration sample (N = 1075)

|  |  | 1 | 2 | 3 | 4 | 5 | 6 | 7 | 8 | 9 | 10 | 11 | 12 | 13 | 14 | 15 | 16 | 17 | 18 | 19 | 20 | 21 | 22 | 23 | 24 | 25 | 26 | 27 | 28 | 29 | 30 | 31 | 32 | 33 | 34 | 35 | 36 | 37 | 38 | 39 | 40 |
| --- | --- | --- | --- | --- | --- | --- | --- | --- | --- | --- | --- | --- | --- | --- | --- | --- | --- | --- | --- | --- | --- | --- | --- | --- | --- | --- | --- | --- | --- | --- | --- | --- | --- | --- | --- | --- | --- | --- | --- | --- | --- |
| 1. Not excited |  | 1.00 |  |  |  |  |  |  |  |  |  |  |  |  |  |  |  |  |  |  |  |  |  |  |  |  |  |  |  |  |  |  |  |  |  |  |  |  |  |  |  |
| 1. Feeling sad |  | .34 | 1.00 |  |  |  |  |  |  |  |  |  |  |  |  |  |  |  |  |  |  |  |  |  |  |  |  |  |  |  |  |  |  |  |  |  |  |  |  |  |  |
| 1. Nothing nice |  | .43 | .45 | 1.00 |  |  |  |  |  |  |  |  |  |  |  |  |  |  |  |  |  |  |  |  |  |  |  |  |  |  |  |  |  |  |  |  |  |  |  |  |  |
| 1. Down and depressed |  | .33 | .71 | .41 | 1.00 |  |  |  |  |  |  |  |  |  |  |  |  |  |  |  |  |  |  |  |  |  |  |  |  |  |  |  |  |  |  |  |  |  |  |  |  |
| 1. Hated myself |  | .28 | .55 | .39 | .57 | 1.00 |  |  |  |  |  |  |  |  |  |  |  |  |  |  |  |  |  |  |  |  |  |  |  |  |  |  |  |  |  |  |  |  |  |  |  |
| 1. I was no good |  | .33 | .55 | .38 | .57 | .66 | 1.00 |  |  |  |  |  |  |  |  |  |  |  |  |  |  |  |  |  |  |  |  |  |  |  |  |  |  |  |  |  |  |  |  |  |  |
| 1. Not doing anything |  | .33 | .45 | .49 | .46 | .42 | .45 | 1.00 |  |  |  |  |  |  |  |  |  |  |  |  |  |  |  |  |  |  |  |  |  |  |  |  |  |  |  |  |  |  |  |  |  |
| 1. Nothing would work |  | .32 | .53 | .44 | .56 | .55 | .60 | .49 | 1.00 |  |  |  |  |  |  |  |  |  |  |  |  |  |  |  |  |  |  |  |  |  |  |  |  |  |  |  |  |  |  |  |  |
| 1. Enjoyed nothing |  | .42 | .47 | .52 | .49 | .42 | .41 | .51 | .47 | 1.00 |  |  |  |  |  |  |  |  |  |  |  |  |  |  |  |  |  |  |  |  |  |  |  |  |  |  |  |  |  |  |  |
| 1. Life was terrible |  | .36 | .60 | .46 | .62 | .67 | .63 | .50 | .64 | .50 | 1.00 |  |  |  |  |  |  |  |  |  |  |  |  |  |  |  |  |  |  |  |  |  |  |  |  |  |  |  |  |  |  |
| 1. Nothing fun |  | .41 | .45 | .49 | .43 | .39 | .40 | .49 | .44 | .69 | .47 | 1.00 |  |  |  |  |  |  |  |  |  |  |  |  |  |  |  |  |  |  |  |  |  |  |  |  |  |  |  |  |  |
| 1. Felt worthless |  | .31 | .57 | .37 | .62 | .66 | .70 | .41 | .62 | .45 | .69 | .45 | 1.00 |  |  |  |  |  |  |  |  |  |  |  |  |  |  |  |  |  |  |  |  |  |  |  |  |  |  |  |  |
| 1. Hated life |  | .36 | .57 | .45 | .59 | .70 | .62 | .45 | .58 | .49 | .79 | .48 | .73 | 1.00 |  |  |  |  |  |  |  |  |  |  |  |  |  |  |  |  |  |  |  |  |  |  |  |  |  |  |  |
| 1. Fool of myself |  | .19 | .30 | .20 | .34 | .34 | .36 | .20 | .32 | .22 | .33 | .21 | .37 | .31 | 1.00 |  |  |  |  |  |  |  |  |  |  |  |  |  |  |  |  |  |  |  |  |  |  |  |  |  |  |
| 1. Lump in throat |  | .22 | .32 | .26 | .35 | .32 | .32 | .30 | .33 | .29 | .35 | .27 | .34 | .29 | .30 | 1.00 |  |  |  |  |  |  |  |  |  |  |  |  |  |  |  |  |  |  |  |  |  |  |  |  |  |
| 1. Shaky hands |  | .18 | .37 | .24 | .37 | .32 | .35 | .32 | .30 | .24 | .32 | .26 | .34 | .30 | .26 | .39 | 1.00 |  |  |  |  |  |  |  |  |  |  |  |  |  |  |  |  |  |  |  |  |  |  |  |  |
| 1. Secretly afraid |  | .24 | .47 | .27 | .48 | .47 | .47 | .32 | .39 | .29 | .44 | .28 | .42 | .41 | .37 | .33 | .44 | 1.00 |  |  |  |  |  |  |  |  |  |  |  |  |  |  |  |  |  |  |  |  |  |  |  |
| 1. Scared by situations |  | .19 | .35 | .22 | .38 | .33 | .38 | .34 | .37 | .24 | .36 | .25 | .35 | .31 | .32 | .28 | .35 | .50 | 1.00 |  |  |  |  |  |  |  |  |  |  |  |  |  |  |  |  |  |  |  |  |  |  |
| 1. Heart beating |  | .19 | .35 | .31 | .38 | .33 | .35 | .36 | .34 | .25 | .37 | .28 | .36 | .35 | .26 | .38 | .47 | .41 | .39 | 1.00 |  |  |  |  |  |  |  |  |  |  |  |  |  |  |  |  |  |  |  |  |  |
| 1. Dry mouth |  | .23 | .29 | .28 | .27 | .24 | .26 | .26 | .28 | .23 | .24 | .29 | .27 | .21 | .18 | .31 | .34 | .26 | .20 | .36 | 1.00 |  |  |  |  |  |  |  |  |  |  |  |  |  |  |  |  |  |  |  |  |
| 1. Nervous |  | .26 | .56 | .35 | .55 | .56 | .57 | .41 | .50 | .44 | .56 | .41 | .51 | .51 | .30 | .37 | .40 | .49 | .41 | .44 | .32 | 1.00 |  |  |  |  |  |  |  |  |  |  |  |  |  |  |  |  |  |  |  |
| 1. Terrified |  | .27 | .49 | .32 | .47 | .46 | .53 | .39 | .44 | .40 | .48 | .40 | .46 | .45 | .27 | .31 | .42 | .50 | .40 | .46 | .28 | .58 | 1.00 |  |  |  |  |  |  |  |  |  |  |  |  |  |  |  |  |  |  |
| 1. Panic |  | .22 | .47 | .25 | .49 | .45 | .47 | .37 | .47 | .36 | .44 | .36 | .49 | .41 | .27 | .41 | .47 | .49 | .44 | .47 | .33 | .58 | .58 | 1.00 |  |  |  |  |  |  |  |  |  |  |  |  |  |  |  |  |  |
| 1. Trouble breathing |  | .21 | .39 | .27 | .39 | .36 | .36 | .35 | .34 | .32 | .38 | .30 | .36 | .32 | .23 | .38 | .38 | .33 | .33 | .47 | .35 | .40 | .40 | .47 | 1.00 |  |  |  |  |  |  |  |  |  |  |  |  |  |  |  |  |
| 1. Dizzy |  | .21 | .35 | .26 | .35 | .35 | .33 | .35 | .29 | .35 | .35 | .35 | .33 | .35 | .19 | .30 | .37 | .31 | .25 | .42 | .35 | .41 | .35 | .42 | .42 | 1.00 |  |  |  |  |  |  |  |  |  |  |  |  |  |  |  |
| 1. Sweaty hands |  | .17 | .28 | .28 | .32 | .24 | .27 | .22 | .27 | .25 | .27 | .22 | .30 | .26 | .22 | .25 | .35 | .28 | .26 | .35 | .34 | .32 | .35 | .36 | .27 | .29 | 1.00 |  |  |  |  |  |  |  |  |  |  |  |  |  |  |
| 1. Scared no reason |  | .24 | .48 | .30 | .52 | .43 | .46 | .40 | .45 | .38 | .49 | .39 | .48 | .45 | .29 | .31 | .37 | .46 | .41 | .45 | .27 | .51 | .52 | .51 | .42 | .42 | .34 | 1.00 |  |  |  |  |  |  |  |  |  |  |  |  |  |
| 1. Difficulty relaxing |  | .23 | .35 | .24 | .42 | .34 | .36 | .31 | .37 | .28 | .34 | .24 | .34 | .33 | .27 | .28 | .31 | .32 | .34 | .28 | .17 | .33 | .28 | .38 | .29 | .27 | .20 | .31 | 1.00 |  |  |  |  |  |  |  |  |  |  |  |  |
| 1. Couldn’t stop thinking |  | .15 | .25 | .18 | .27 | .20 | .27 | .22 | .28 | .14 | .25 | .17 | .23 | .25 | .28 | .22 | .24 | .27 | .36 | .22 | .03 | .22 | .16 | .24 | .17 | .16 | .14 | .23 | .40 | 1.00 |  |  |  |  |  |  |  |  |  |  |  |
| 1. Stressing lots |  | .22 | .38 | .22 | .46 | .37 | .39 | .34 | .41 | .27 | .35 | .28 | .39 | .36 | .26 | .30 | .35 | .42 | .39 | .31 | .17 | .36 | .32 | .42 | .29 | .25 | .22 | .31 | .51 | .55 | 1.00 |  |  |  |  |  |  |  |  |  |  |
| 1. Little things upset |  | .23 | .48 | .25 | .51 | .41 | .49 | .36 | .44 | .26 | .44 | .28 | .42 | .43 | .28 | .32 | .32 | .47 | .37 | .32 | .22 | .45 | .39 | .44 | .32 | .29 | .19 | .45 | .38 | .32 | .48 | 1.00 |  |  |  |  |  |  |  |  |  |
| 1. Easily irritated |  | .25 | .38 | .25 | .43 | .35 | .39 | .34 | .41 | .26 | .36 | .29 | .34 | .33 | .29 | .26 | .26 | .34 | .28 | .32 | .25 | .33 | .29 | .37 | .28 | .25 | .20 | .33 | .39 | .31 | .42 | .46 | 1.00 |  |  |  |  |  |  |  |  |
| 1. Annoyed |  | .17 | .26 | .29 | .28 | .22 | .26 | .31 | .30 | .24 | .26 | .30 | .21 | .23 | .21 | .17 | .24 | .24 | .27 | .29 | .31 | .33 | .27 | .28 | .30 | .26 | .26 | .27 | .27 | .19 | .22 | .31 | .44 | 1.00 |  |  |  |  |  |  |  |
| 1. Overreacting |  | .22 | .42 | .30 | .46 | .44 | .47 | .38 | .48 | .29 | .43 | .31 | .46 | .43 | .32 | .31 | .32 | .40 | .35 | .35 | .27 | .42 | .35 | .43 | .35 | .30 | .27 | .40 | .42 | .33 | .43 | .52 | .49 | .40 | 1.00 |  |  |  |  |  |  |
| 1. Frustrating to wait |  | .18 | .28 | .27 | .28 | .28 | .28 | .41 | .33 | .31 | .29 | .33 | .25 | .28 | .19 | .24 | .28 | .25 | .30 | .33 | .27 | .33 | .32 | .32 | .34 | .30 | .28 | .29 | .29 | .23 | .31 | .32 | .39 | .41 | .37 | 1.00 |  |  |  |  |  |
| 1. Tense uptight |  | .27 | .43 | .32 | .51 | .46 | .47 | .39 | .49 | .42 | .47 | .37 | .48 | .45 | .32 | .41 | .42 | .44 | .39 | .47 | .27 | .46 | .44 | .58 | .42 | .37 | .32 | .43 | .46 | .37 | .51 | .43 | .45 | .26 | .49 | .35 | 1.00 |  |  |  |  |
| 1. Hated stopping |  | .13 | .22 | .19 | .20 | .21 | .23 | .28 | .27 | .22 | .26 | .23 | .22 | .22 | .18 | .17 | .21 | .25 | .26 | .26 | .24 | .26 | .25 | .26 | .28 | .28 | .30 | .27 | .24 | .18 | .18 | .21 | .27 | .36 | .30 | .40 | .26 | 1.00 |  |  |  |
| 1. Couldn’t calm down |  | .26 | .49 | .34 | .49 | .45 | .49 | .44 | .50 | .39 | .52 | .41 | .47 | .46 | .29 | .28 | .36 | .41 | .41 | .39 | .26 | .50 | .45 | .54 | .41 | .40 | .32 | .50 | .38 | .30 | .43 | .47 | .35 | .37 | .46 | .33 | .48 | .34 | 1.00 |  |  |
| 1. Easily annoyed |  | .22 | .31 | .30 | .38 | .34 | .38 | .40 | .42 | .30 | .39 | .34 | .33 | .35 | .24 | .24 | .27 | .28 | .30 | .31 | .26 | .39 | .32 | .33 | .29 | .27 | .26 | .34 | .34 | .30 | .39 | .42 | .61 | .54 | .52 | .47 | .42 | .39 | .46 | 1.00 |  |
| 1. Upset easily |  | .25 | .52 | .30 | .57 | .44 | .51 | .41 | .51 | .35 | .50 | .35 | .48 | .48 | .31 | .33 | .35 | .44 | .33 | .36 | .24 | .48 | .44 | .49 | .34 | .33 | .28 | .50 | .39 | .29 | .47 | .62 | .51 | .36 | .53 | .35 | .50 | .28 | .54 | .56 | 1.00 |

Note. All correlations are significant at *p* < .0001
