# Supplementary material for: Development and Psychometric Properties of the DASS-Youth (DASS-Y): An Extension of the Depression Anxiety Stress Scales (DASS) to Adolescents and Children
Source: Front Psychol. 2022 Apr 14;13:766890. doi: 10.3389/fpsyg.2022.766890 (PMC9047499; doi:10.3389/fpsyg.2022.766890)
Supplement: Supplementary file 2 [file Table_2.DOCX]

Supplementary Table 2. Factor loadings for all 40 draft DASS-Y items in the calibration sample (n = 1075) in high school students and primary school students separately.

| High school students (n = 640) | | | |
| --- | --- | --- | --- |
|  | Depression | Anxiety | Stress |
| Life was terrible | .887 |  |  |
| Hated life | .872 |  |  |
| Felt worthless | .864 |  |  |
| Feeling sad | .849 |  |  |
| Down and depressed | .848 |  |  |
| Hated myself | .814 |  |  |
| I was no good | .802 |  |  |
| Nothing would work | .775 |  |  |
| Enjoyed nothing | .715 |  |  |
| Nothing fun | .677 |  |  |
| Not doing anything | .641 |  |  |
| Nothing nice | .625 |  |  |
| Not excited | .504 |  |  |
| Panic |  | .790 |  |
| Nervous |  | .781 |  |
| Terrified |  | .767 |  |
| Scared no reason |  | .733 |  |
| Secretly afraid |  | .699 |  |
| Heart beating |  | .687 |  |
| Trouble breathing |  | .685 |  |
| Shaky hands |  | .657 |  |
| Dizzy |  | .631 |  |
| Lump in throat |  | .629 |  |
| Scared by situations |  | .620 |  |
| Dry mouth |  | .533 |  |
| Fool of myself |  | .506 |  |
| Sweaty hands |  | .484 |  |
| Upset easily |  |  | .801 |
| Overreacting |  |  | .755 |
| Tense uptight |  |  | .753 |
| Couldn’t calm down |  |  | .743 |
| Easily annoyed |  |  | .738 |
| Little things upset |  |  | .729 |
| Easily irritated |  |  | .720 |
| Stressing lots |  |  | .674 |
| Difficulty relaxing |  |  | .650 |
| Annoyed |  |  | .577 |
| Frustrating to wait |  |  | .571 |
| Couldn’t stop thinking |  |  | .525 |
| Hated stopping |  |  | .445 |

Supplementary Table 2. (continued)

| Primary school students (n = 435) | | | |
| --- | --- | --- | --- |
|  | Depression | Anxiety | Stress |
| Life was terrible | .802 |  |  |
| Hated life | .772 |  |  |
| Felt worthless | .730 |  |  |
| I was no good | .727 |  |  |
| Hated myself | .705 |  |  |
| Nothing would work | .700 |  |  |
| Down and depressed | .630 |  |  |
| Feeling sad | .576 |  |  |
| Not doing anything | .571 |  |  |
| Nothing fun | .515 |  |  |
| Nothing nice | .497 |  |  |
| Enjoyed nothing | .496 |  |  |
| Not excited | .371 |  |  |
| Panic |  | .703 |  |
| Nervous |  | .685 |  |
| Terrified |  | .638 |  |
| Scared no reason |  | .633 |  |
| Secretly afraid |  | .630 |  |
| Heart beating |  | .601 |  |
| Shaky hands |  | .537 |  |
| Scared by situations |  | .526 |  |
| Trouble breathing |  | .491 |  |
| Sweaty hands |  | .479 |  |
| Dizzy |  | .459 |  |
| Dry mouth |  | .380 |  |
| Lump in throat |  | .377 |  |
| Fool of myself |  | .358 |  |
| Upset easily |  |  | .682 |
| Couldn’t calm down |  |  | .634 |
| Overreacting |  |  | .633 |
| Easily annoyed |  |  | .626 |
| Stressing lots |  |  | .626 |
| Little things upset |  |  | .623 |
| Tense uptight |  |  | .600 |
| Easily irritated |  |  | .581 |
| Frustrating to wait |  |  | .509 |
| Hated stopping |  |  | .480 |
| Annoyed |  |  | .476 |
| Difficulty relaxing |  |  | .464 |
| Couldn’t stop thinking |  |  | .408 |
